# Comparing Simplification Strategies for the Skeletal Muscle Proteome

**DOI:** 10.3390/proteomes4010010

**Published:** 2016-03-02

**Authors:** Bethany Geary, Iain S. Young, Phillip Cash, Phillip D. Whitfield, Mary K. Doherty

**Affiliations:** 1 Division of Health Research, University of the Highlands and Islands, Inverness IV2 3JH, UK; beth.geary@uhi.ac.uk (B.G.); phil.whitfield@uhi.ac.uk (P.D.W.); 2 Institute of Integrative Biology, University of Liverpool, Liverpool L69 7ZB, UK; isyoung@liverpool.ac.uk; 3 Division of Applied Medicine, University of Aberdeen, Aberdeen AB25 2ZD, UK; p.cash@abdn.ac.uk

**Keywords:** FASP, OFFGEL, protein equalization, proteome simplification, ProteoMiner, skeletal muscle

## Abstract

Skeletal muscle is a complex tissue that is dominated by the presence of a few abundant proteins. This wide dynamic range can mask the presence of lower abundance proteins, which can be a confounding factor in large-scale proteomic experiments. In this study, we have investigated a number of pre-fractionation methods, at both the protein and peptide level, for the characterization of the skeletal muscle proteome. The analyses revealed that the use of OFFGEL isoelectric focusing yielded the largest number of protein identifications (>750) compared to alternative gel-based and protein equalization strategies. Further, OFFGEL led to a substantial enrichment of a different sub-population of the proteome. Filter-aided sample preparation (FASP), coupled to peptide-level OFFGEL provided more confidence in the results due to a substantial increase in the number of peptides assigned to each protein. The findings presented here support the use of a multiplexed approach to proteome characterization of skeletal muscle, which has a recognized imbalance in the dynamic range of its protein complement.

## 1. Introduction

Skeletal muscle is a complex tissue that is dominated by the presence of a few abundant proteins with myosin and actin constituting almost 40% of the total protein pool of the muscle tissue [1]. These proteins are found predominantly in the contractile assembly. Whilst this can be easily removed through the use of low ionic strength conditions, in which it is insoluble [2], the remaining soluble protein fraction is also highly complex, with a wide dynamic range of protein concentrations that cover several orders of magnitude.

LC-MS/MS has emerged as a key technology for profiling protein expression patterns in skeletal muscle [3,4] but even the most advanced platforms are unable to fully cover the dynamic range of protein abundance found in muscle [5]. As a result, there can be an over representation of a sub-set of proteins that dominate analyses and proteins of low abundance may not be detected, leading to the incomplete characterization of the proteome and ultimately a loss of biological information. Proteomic investigations of skeletal muscle therefore represent a considerable analytical challenge. A possible means of addressing this issue is to perform some level of simplification prior to mass spectrometric analysis [6].

A number of proteome simplification strategies are available. These include sub-cellular fractionation [7] and techniques such as gel electrophoresis, isoelectric focusing, and ion exchange chromatography that involve the stringent separation of protein (or peptide) mixtures [8]. Another approach is immunodepletion, which utilizes antibodies to selectively remove the most abundant proteins as a means of enhancing the detection of lower abundance proteins [9]. The latter approach is typically employed for proteomic studies of body fluids, e.g., blood plasma or serum, but concerns exist about the use of depletion steps since low abundant proteins may be simultaneously removed if they interact with the target proteins [10,11]. A further simplification strategy involves the use of protein equalization technologies [12,13] that employ combinatorial peptide ligand libraries to create a more uniform distribution of protein abundance and thereby reduce the dynamic range of protein mixtures in biological samples. This methodology has been employed with an increasing number of sample types [14,15,16].

In this study, we have compared a variety of simplification strategies for the characterization of the skeletal muscle proteome. We have applied these methodologies to the analysis of skeletal muscle from common carp (*Cyprinus carpio*), a natural model system that is used to study the molecular changes underpinning muscle plasticity [17,18,19]. The experimental strategies, which were evaluated in terms of the number of identified proteins and the confidence of the identifications, were able to reduce the complexity and dynamic range of the protein composition improving coverage of the muscle proteome.

## 2. Experimental Section

### 2.1. Experimental Animals

In line with the principles of the 3Rs (Replacement, Reduction, and Refinement) skeletal muscle was used from common carp that been sampled as part of previous studies focused on the proteome dynamics of muscle [20,21]. Carp (average weight 19 g/fish) were maintained in the University of Liverpool aquarium at 25 °C ± 0.5 °C (at pH 6.5–7.0) on a 16 h light: 8 h dark photoperiod. The carp were fed an experimental diet, in which 50% of the l-leucine in the diet (that proportion added as crystalline amino acid) was replaced with [^2^H_7_] l-leucine (98% purity) (Cambridge Isotope Laboratories, Tewksbury, MA, USA). Fish were killed in accordance with UK Home Office Schedule One regulations. All tissues were stored at −80 °C prior to analysis.

### 2.2. Sample Preparation

The skeletal muscle samples (*n* = 3; approximately 300–400 mg wet weight of tissue) were mechanically homogenized in 2.5 mL of 20 mM sodium phosphate buffer (pH 7.4) containing Complete Protease Inhibitors (Roche, Lewes, UK). The homogenate was centrifuged at 15,000× *g* at 4 °C for 45 min and the supernatant was removed. The remaining pellet was re-suspended in 1 mL of sodium phosphate buffer, re-homogenized, and centrifuged and the supernatant was combined with the supernatant set aside in the previous step. The protein concentration of each sample was determined using the Coomassie Plus Protein Assay (Pierce Biotechnology, Rockford, IL, USA).

### 2.3. 1-D SDS-PAGE

The soluble proteins (20 µg) from skeletal muscle samples were separated by 1-D SDS-PAGE using a Mini-Protean Tetra system (Bio-Rad Laboratories Ltd, Hemel Hempstead, UK). Samples were electrophoresed at a constant potential of 200 V through a 15% *w*/*v* polyacrylamide resolving gel with a 4% *w*/*v* stacking gel. Samples were incubated at 95 °C for 5 min in a reducing buffer (125 mM Tris-HCl; 140 mM SDS; 20% *v*/*v* glycerol; 200 mM DTT and 30 mM bromophenol blue) prior to loading. Gels were stained with Coomassie Blue. Gel lanes were cut into 12 slices and each slice placed in deionised water (50 µL). The water was then removed and the gel piece was treated with destain solution (10 µL of acetonitrile/100 mM ammonium bicarbonate 1:1 *v*/*v*). The protein disulphide bonds were reduced by the addition of DTT and alkylated by iodoacetamide. Each gel slice was dehydrated in acetonitrile. Trypsin (Roche) (0.2 µg/µL in 50 mM acetic acid) was added at a ratio of protein:trypsin 50:1 and the digestion allowed to proceed overnight at 37 °C. The peptides were then extracted from the gel by addition of acetonitrile and then dried with a MiVac centrifugal vacuum concentrator (Genevac, Ipswich, UK) prior to resuspension in 50% *v/v* methanol.

### 2.4. ProteoMiner Protein Equalization

200 μL of protein homogenate was concentrated to 40 mg/mL before application to the ProteoMiner low-yield enrichment kit (Bio-Rad Laboratories Ltd). Briefly, the column was centrifuged to remove storage material and washed twice with 200 μL of wash buffer (PBS pH 7.4). The protein homogenate (200 μL) was then introduced to the column, which was rotated overnight at room temperature for sample binding. The column was washed with wash buffer and then deionised water before elution using 30 μL of boiled 1-D SDS-PAGE loading buffer. All fractions were subjected to 1-D SDS-PAGE analysis.

### 2.5. OFFGEL Isoelectric Focusing

250 μg protein was mixed with OFFGEL 1.25× buffer (8.4 M urea, 2.4 M thiourea, 0.08 M DTT, 12% *v*/*v* glycerol, 600 µL of ampholyte buffer pH 3–10) prior to loading onto rehydrated IPG strips (13 mm, pH 3–10, GE Healthcare, Little Chalfont, UK) and used on an OFFGEL 3100 fractionator isoelectric focusing unit (Agilent, Santa Clara, CA, USA). Samples were run for 20 kVh with a voltage limit of 8000 V and a current limit of 50 µA. The 12 liquid fractions were removed from the wells and proteins extracted using chloroform-methanol precipitation followed by de-salting using StageTips (Thermo Fisher, Waltham, MA, USA) prior to 1-D SDS-PAGE analysis.

### 2.6. Filter Aided Sample Preparation (FASP)

Skeletal muscle samples (*n* = 3) were homogenized in 400 µL of 4% *w*/*v* SDS, 100 mM Tris/HCL pH 7.6, 0.1 M DTT and incubated at 95 °C for 3 min then centrifuged at 14,000× *g* for 45 min at 4 °C. 30 µL of concentrated sample was added to 200 µL of urea buffer (8 M urea in 0.1 M Tris-HCl, pH 8.5) in a 10 kDa centrifugal filter unit (Millipore, Billerica, MA, USA) and centrifuged at 14,000× *g* for 15 min. A further 200 µL of urea buffer was then added to the filter unit and centrifuged at 14,000× *g* for 15 min. The effluent was discarded and 100 µL of 0.05 M iodoacetamide solution was then added. The samples were thoroughly mixed prior to incubation at room temperature for 20 min and then centrifuged for 10 min at 14,000× *g*. Urea buffer (100 µL) was added and centrifuged for 15 min at 14,000× *g*. This step was repeated twice. 100 µL of 0.05 M ammonium bicarbonate was then added and centrifuged at 14,000× *g* for 10 min. This step was repeated a further two times. All effluent was discarded. 40 µL of ammonium bicarbonate containing trypsin (trypsin: protein ratio 100:1) was then added to the filter unit, which was incubated at 37 °C overnight after which the filter units were then transferred to fresh collection tubes and centrifuged at 14,000× *g* for 10 min. 40 µL of ammonium bicarbonate was added prior to a final centrifugation at 14,000× *g* for 10 min. The resulting filtrates were acidified with trifluoroacetic acid to a final concentration of 0.1% *v*/*v*. The peptides were desalted using StageTips and then separated by OFFGEL isoelectric focusing with targets of 20 kVh with limits of 8 kV, 50 µA and 200 mW. Samples were then buffer exchanged using StageTips to a final methanol concentration of 50% *v*/*v* prior to LC-MS/MS analysis.

### 2.7. Analysis of Peptides by LC-MS/MS

Peptide analysis was performed in positive ion mode using a LTQ-Orbitrap XL LC-MS^n^ mass spectrometer (Thermo Fisher, Hemel Hempsted, UK) equipped with a nanospray source and coupled to a nanoAcquity UPLC system (Waters, Manchester, UK). The samples were initially concentrated on a BEH C18 trapping column (Waters). The peptides were then separated on a BEH C18 nanocolumn (1.7 µm, 75 µm × 250 mm, Waters) at a flow rate of 400 nL/min. Solvent A comprised 0.1% formic acid in water and solvent B comprised 0.1% formic acid in acetonitrile. Peptides were separated using a gradient of 10%–40% B over 120 min. Acquisition was in data-dependent mode over *m*/*z* 300–2000 using a precursor ion resolution of 30,000. The top 10 precursor ions were automatically isolated and fragmented using collision induced dissociation (CID) with a relative collision energy of 35%. Dynamic exclusion was enabled. A repeat count of 1 was used with a repeat duration of 30 s and exclusion duration of 60 s.

### 2.8. Protein Identifications

Data were processed using MaxQUANT v1.2.2.5 with the Andromeda search engine [22,23]. Data were searched against the UniProt *Danio rerio* IPI database v3.6.7). Search parameters of a precursor ion mass tolerance of 20 ppm and fragment ion mass tolerance of 0.5 Da, two missed cleavages, a maximum 1% false discovery rate were used for both protein and peptide identification. Fixed modifications of carbamidomethylation of cysteine and the variable modifications of methionine oxidation and acetylation of the protein *N*-terminus were used in all searches. An additional parameter, coded as a pseudo post-translational modification was included to search for peptides containing [^2^H_7_]-leucine. Protein identifications were made from a minimum of two peptides per protein including at least one unique peptide. For proteins that were described as “novel” or “hypothetical”, a BLAST search was performed in order to ascertain the identity of homologous proteins.

### 2.9. Gene Ontology

Functional categories of proteins from the gene ontology consortium (http://geneontology.org/) were assigned using the PANTHER (http://pantherdb.org/) gene list analysis research tool [24,25]. The complete zebrafish genome was used as a reference. Enrichment of the different GO terms was also performed using PANTHER with the data Bonferroni corrected for multiple testing.

## 3. Results and Discussion

### 3.1. Experimental Design

**Figure 1:**
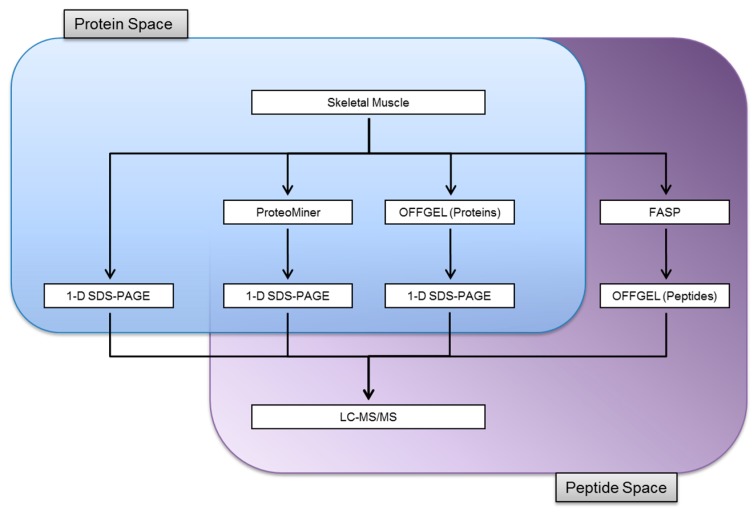
A multiplexed approach to characterising the skeletal muscle proteome skeletal muscle from common carp was homogenized and subjected to a range of separation techniques prior to mass spectrometric analyses. The soluble protein fraction was either separated by 1-D SDS-PAGE or simplified by either ProteoMiner protein equalization or OFFGEL isoelectric focusing before the eluted fractions were analysed by 1-D SDS-PAGE. A further strategy analysed the whole protein homogenate by sampling directly in the peptide space. Proteins were digested in-solution prior to OFFGEL fractionation.

We compared the identifications of soluble proteins obtained from skeletal muscle by 1-D SDS-PAGE with OFFGEL isoelectric focusing and the enrichment of low abundance proteins by ProteoMiner chemical hexapeptide libraries. The whole skeletal muscle proteome was also fractionated at the peptide level through the use of FASP coupled to OFFGEL separation. A schematic of the experimental workflow is presented in Figure 1. The experiments were performed on skeletal muscle obtained from common carp that had been administered a diet containing deuterated leucine. Whilst the efficient use of these tissues eliminated the need to harvest additional fish, it also offered the benefit that peptides containing stable isotope labelled leucine residues provided a further level of confidence for protein identifications. The full genome of common carp is available although it still lacks detailed annotation. As a result, the carp muscle proteins were identified by matching to zebrafish (*Danio rerio*), that has been sequenced and extensively annotated. This approach was facilitated by the close taxonomic relationship of carp and zebrafish, which are both cyprinids.

### 3.2. Protein Identifications in Skeletal Muscle by 1-D SDS-PAGE

**Figure 2:**
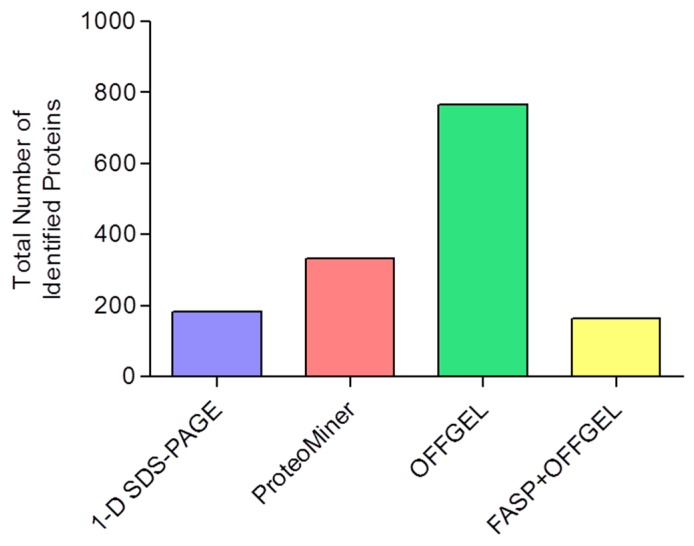
Protein identifications following sample pre-fractionation. The number of proteins identified following each fractionation protocol was determined. Data shown was determined from three biological replicates for each technique.

We have previously used 1-D SDS-PAGE in conjunction with peptide mass fingerprinting and *de novo* sequencing to identify the abundant proteins in carp skeletal muscle [26]. Using this approach, it was only possible to confidently identify 24 proteins. Additional separation of the proteins by 2D-PAGE did not significantly increase the number of proteins identified. The protein composition was found to largely comprise creatine kinase isoforms and fragments and enzymes of the glycolytic pathway. In the current study, with a GeLC-MS/MS approach, the average number of identifications for each biological replicate was 120 ± 1 (Table 1). Across the three biological replicates, a total of 182 carp skeletal muscle proteins were confidently assigned (Figure 2 and Supplemental Table S1) indicating that increasing the number of replicates permits a more extensive coverage of the proteome.

**Figure 3:**
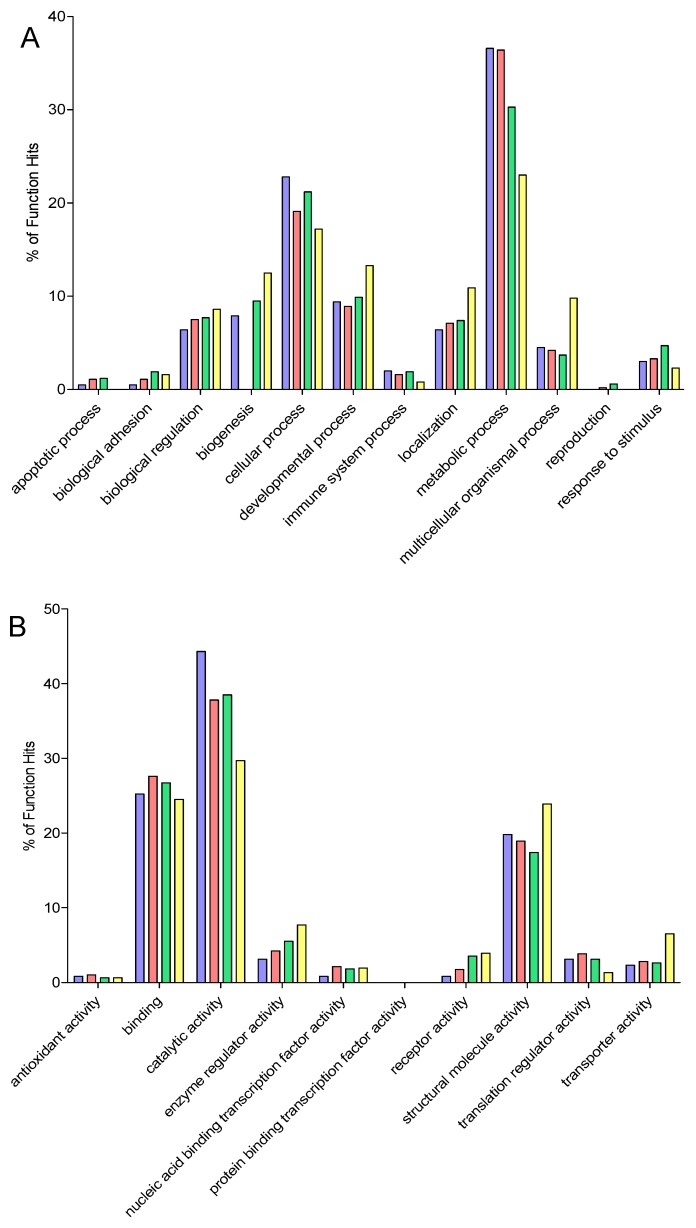

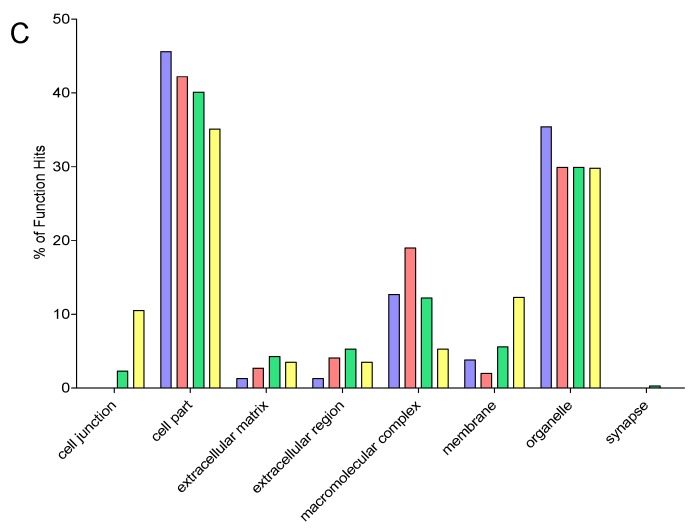
Gene ontology analysis. Gene ontology analysis was performed using the PANTHER software. Proteins were grouped according to (**A**) biological function, (**B**) molecular function and (**C**) cellular component. 1-D SDS-PAGE is represented by blue bars, ProteoMiner by red, OFFGEL by green and FASP by yellow.

Gene ontology (GO) analysis (Figure 3 and Supplemental Table S2) revealed that proteins were predominantly metabolic (37%, GO Biological Process) followed by cellular process (23%) and developmental process (10%). The major metabolic functions of identified proteins were catalytic activity (44%), binding (25%), and structural molecule activity (20%). Proteins were largely intracellular and 35% were found to correspond to organelles. Almost 13% of identified proteins were found to be components of macromolecular complexes. There were a wide variety of protein classes identified (23 in total), which included cytoskeletal proteins (17%) and a range of enzymes including transferases (11%), hydrolases (11%), and oxidoreductases (10%). Enrichment analysis showed that glycolysis, TCA cycle, carbohydrate (including monosaccharide) metabolic processes, muscle contraction, and muscle organ development were all significantly enriched by more than five-fold compared to the complete *Danio rerio* genome. Similarly, the molecular functions of actin binding, isomerase activity, cytoskeletal protein binding, and structural constituents of the cytoskeleton together with the cellular compartments related to the cytoskeleton and tubulin complexes were also enriched.

### 3.3. Proteome Equalization Increases the Number of Protein Identifications

The soluble muscle proteins were subjected to protein equalization and compared with unfractionated skeletal muscle. 1-D SDS-PAGE revealed that after treatment with the ProteoMiner system there was a substantial enrichment of the low-abundance proteins (Supplemental Figure S1). This finding was reflected in the subsequent LC-MS/MS analysis, that led to a substantial increase in protein identifications (mean = 209 ± 15; Table 1). The total number of proteins identified over the three biological replicates was 334 (Figure 2 and Supplemental Table 1). A study by Rivers *et al.* [15] achieved a similar number of protein identifications from chicken skeletal muscle (>350 unique proteins detected) using a protein equalization strategy. Whilst this approach was effective in reducing the most abundant proteins, the authors indicated that there was a different bias in the proteins detected, particularly when different ionic strength buffers were used during sample loading.

In the GO analysis of the ProteoMiner simplified protein fractions (Figure 3 and Supplemental Table S2), the major molecular functions, biological process, and cellular compartment were similar to the 1-D SDS-PAGE analysis. However, there was a difference in the protein class distribution with nucleic acid binding proteins the predominant protein class (15%). Enrichment analysis of the proteins identified following ProteoMiner revealed that protein equalization covered a greater number of biological processes with the addition of protein complex assembly and biogenesis, protein folding, regulation of translation, and glycogen metabolic processes. However, muscle organ development and muscle contraction were significantly enriched to a lesser extent than in 1-D SDS-PAGE. Cellular localization enrichment revealed three components that were more than five-fold enriched. These were tubulin complex, ribosome, and cytosol. Nine specific pathways were significantly enriched including TCA cycle, glycolysis, cytoskeletal regulation by Rho GTPase, pyruvate metabolism, cell cycle, UPS, and both FGF and EGF signalling pathways.

### 3.4. Extending Proteome Coverage Through In-Solution Isoelectric Focusing

We also sought to pre-fractionate the protein complement of skeletal muscle through the use of OFFGEL. Separated protein fractions were recovered in the liquid phase and subjected to 1-D SDS-PAGE. The gel analysis displayed a clear increase in the number of observable proteins for each resolved fraction (Supplemental Figure S2) and there was a near four-fold increase in the mean number of identifications to 425 ± 62 proteins (Table 1). The total identified proteins across all biological replicates was 766 (Figure 2 and Supplemental Table S1). This result demonstrates the resolving power of this fractionation strategy, that permits more extensive coverage of the skeletal muscle proteome although it extends sample preparation and requires additional LC-MS/MS run time.

GO analysis of the OFFGEL datasets indicated that the majority of proteins were involved in metabolic processes (30%) and cellular function (21%). Around 10% were involved in both developmental processes and biogenesis (Figure 3 and Supplemental Table S2). Molecular functions and cellular component analysis revealed a similar profile to both 1-D SDS-PAGE and simplification by protein equalization. Twenty-six different protein classes were represented in the OFFGEL separated proteins, although, the major classes were similar to the previous analyses. Enrichment of biological processes revealed eight that were more than five-fold enriched, the majority of which overlapped with the ProteoMiner bead simplification of the proteome. The molecular function enrichment was however somewhat different and included amino acid kinase activity, phosphatase inhibitor activity, aminoacyl-tRNA ligase activity, translation elongation factor activity, phosphatase regulator activity, translation factor activity, nucleic acid binding, translation regulator activity, translation initiation factor activity and isomerase activity. Again, tubulin complex and ribosomal proteins were significantly enriched more than five-fold as were proteins located in the intermediate filament cytoskeleton. Pathway enrichment was similar to both 1-D SDS-PAGE and ProteoMiner simplification approaches, with the addition of the methylmalonyl pathway.

### 3.5. Improving the Confidence of Protein Identifications by FASP

We also evaluated the effect of pre-fractionation at the peptide level. In these experiments skeletal muscle homogenate was digested in solution through FASP. The resultant peptides were then separated by OFFGEL isoelectric focusing and the collected fractions were analysed by LC-MS/MS. Using this approach, the mean number of protein identifications was 118 ± 9 (Table 1), with a total of 163 proteins confidently identified across the biological replicates (Figure 2 and Supplemental Table S1). Analysis of the peptides derived from the FASP analysis alone (prior to OFFGEL separation) resulted in the identification of 75 ± 9 proteins (*n* = 3 replicates), with a total of 91 proteins across the replicates. Although the FASP + OFFGEL and 1-D SDS-PAGE analyses resulted in a similar number of total identifications, there was an overlap of around only 50% (88 proteins) indicating that FASP analysis sampled a different sub-proteome of the skeletal muscle (Supplemental Figure S3).

The GO analysis revealed that a comparable profile with respect to biological process and molecular function was observed (Figure 3 and Supplemental Table S2). Whilst the cellular compartments of the proteins were broadly similar to the other simplification processes, the percentage distributions of the locations were different. Almost 35% of proteins were deemed to be cellular, with 30% attributed to organelles and over 12% to membranes. A further 10% of proteins were located to cell junctions. Twenty-three classes of proteins were identified and again cytoskeletal proteins were the major class although at an increased level of 22%. The next most abundant class was enzyme modulator (9%) and cell junction proteins (8%).

Thirty biological processes were found to be enriched with reference to the zebrafish genome, 18 of which were more than five-fold enriched. These were neuromuscular synaptic transmission, glycolysis, tricarboxylic acid cycle, sensory perception of sound, cytokinesis, muscle contraction, monosaccharide metabolic process, cellular component morphogenesis, generation of precursor metabolites and energy, muscle organ development, cellular component movement, anatomical structure morphogenesis, mitosis, mesoderm development, carbohydrate metabolic process, cellular component organization, cellular component organization or biogenesis, and sensory perception. Similarly, there were 12 biological processes and nine cellular components that were significantly enriched. There were however only four pathways found to be enriched-glycolysis, cytoskeletal regulation by Rho GTPase, nicotinic acetylcholine receptor signaling pathway, and the Wnt signaling pathway.

### 3.6. Comparison of Identification and Overlaps

**Figure 4:**
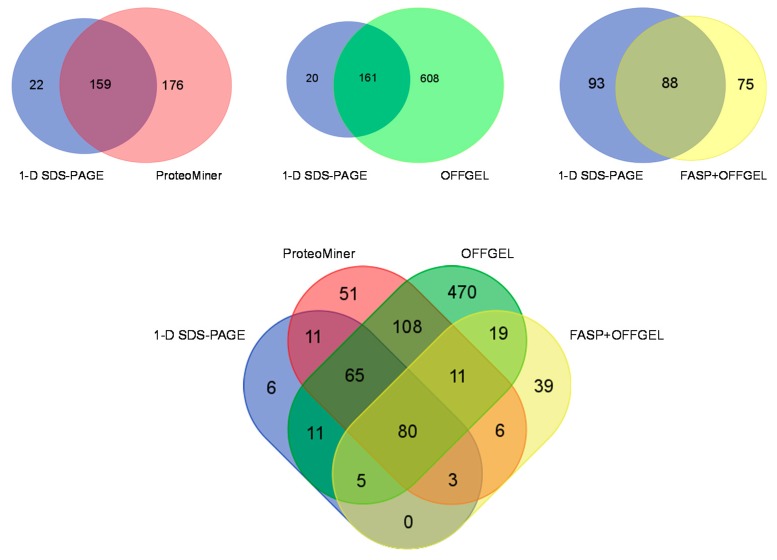
Overlap of Protein Identifications. There were 80 identified proteins common to all experimental approaches. OFFGEL analysis yielded the greatest number of uniquely identified proteins, followed by ProteoMiner, FASP with 1-D SDS-PAGE having the fewest.

The four different simplification strategies provided a different composition of identified proteins. Whilst there was some overlap between the different techniques in each approach, there were a number of proteins that were uniquely identified (Figure 4).

When compared to 1-D SDS-PAGE analysis, simplification by proteome equalization technology yielded 159 proteins that were common to both analyses and 176 unique proteins. The OFFGEL analysis identified the largest number of unique proteins of any of the methodologies and identified all but 20 of those observed in the 1-D SDS-PAGE analysis alone. FASP yielded the lowest number of protein identifications. A FASP strategy together with OFFGEL fractionation has also recently been applied to the deep mining of murine skeletal muscle [27] and single muscle fibre analysis [28]. In our study the FASP analysis involved the use whole muscle homogenate rather than soluble muscle fraction and therefore would have sampled a different sub-proteome of the skeletal muscle. The lower number of protein identifications may result from losses during buffer exchange in the FASP protocol prior to OFFGEL analysis. In view of these results, this approach may need to be further refined for use with fish skeletal muscle. This could be achieved by employing different buffers and desalting methods. However, it should be noted that the mean number of peptides from FASP analysis was much higher than all other strategies at 20.5% compared to between 6% and 7% for 1-D SDS-PAGE, ProteoMiner, and OFFGEL. In total, 80 proteins were identified across all of the different strategies. GO analysis revealed this group was comprised of mostly catalytic activity and structural proteins that are typically present in high abundance in muscle tissue.

Enrichment analysis indicated clear differences in the classifications of the identified proteins. Eight biological processes (anatomical structure morphogenesis, carbohydrate metabolic process, generation of precursor metabolites and energy, glycolysis, metabolic process, monosaccharide metabolic process, muscle contraction, and tricarboxylic acid cycle) were enriched by all methods. Cell communication was only enriched following ProteoMiner simplification, whereas there were 11 categories specifically enriched following both OFFGEL separation and FASP. Only four molecular functions (actin binding, catalytic activity, structural constituent of cytoskeleton, and structural molecule activity) and five cellular compartments (actin cytoskeleton, cytoskeleton, intracellular, organelle, and tubulin complex) were enriched across all analyses. These results indicate that different approaches to sample pre-fractionation in proteomic experiments minimise bias and reinforce the need for a multi-layer approach to proteome characterization. Moreover, an analysis of each analytical approach showed that there was some variation in the proteins identified between technical replicates. This ranged from 46% overlap of identified proteins between OFFGEL experiments up to 66% for both 1D-SDS-PAGE and FASP (Table 2). Further, each approach was assessed in terms of its experimental practicality. For a rapid screen of the most abundant proteins in skeletal muscle, 1D-SDS-PAGE would be appropriate, whereas ProteoMiner provides a robust analysis of lower abundance proteins. OFFGEL enables the most comprehensive profiling of the proteome but is both time and resource intensive and therefore may not be suitable for a large cohort study. Given the larger numbers of peptides identified per protein FASP might be best employed for quantitative studies.

## 4. Conclusions

Proteomic analyses underrepresent low-abundance proteins in a given biological sample. Skeletal muscle is a particularly challenging tissue as its proteome displays a wide dynamic range of protein concentrations. The focus of this study was to compare different simplification strategies aimed at improving the characterization of the protein complement of carp skeletal muscle. Both ProteoMiner and OFFGEL approaches substantially increased the number of identifications compared to the use of 1-D SDS-PAGE alone. With the FASP technique there was an increase in the number of peptides observed per protein, thus improving the confidence with which their identity was assigned. The simplification strategies outlined in this paper provide robust and reproducible means of enhancing the identification of proteins in proteomic studies of skeletal muscle.

## Figures and Tables

**Table 1:** Protein Identifications from Skeletal Muscle.

Technique ^a^	Total Number of Identified Proteins	Mean Number of Identified Proteins	Mean Number of Peptides per Protein	Mean Sequence Coverage (%)
1-D SDS-PAGE	182	120 ± 1	7.5 ± 0.2	20 ± 2
ProteoMiner	334	209 ± 15	7.3 ± 0.4	18 ± 4
OFFGEL	766	425 ± 62	6.4 ± 0.1	17 ± 0.2
FASP+OFFGEL	163	118 ± 9	20.5 ± 0.3	29 ± 1

^a^ *n* = 3 skeletal muscle replicates.

**Table 2:** Comparison of Different Experimental Approaches.

Method	1-D SDS-PAGE	ProteoMiner	OFFGEL	FASP
Time from tissue homogenisation to protein digest	2 days	3 days	4 days+	3 days
Number of protein digests per tissue sample	12	12	144	12
Overlap between replicate identifications	66	57	46	66
Total experimental time	4 days	5 days	25 days	4 days
File size demands	Low	Low	Very high	Low

## Supplementary Materials

The following are available online at http://www.mdpi.com/2227-7382/4/1/10/s1, Figure S1: Visualisation of ProteoMiner Bead Equalisation. Proteins were subjected to equalisation using bead technology in order to reduce the dominance of highly abundant proteins. Panel A shows the pre-fractionated skeletal muscle soluble proteome with the simplified proteome in Panel B; Figure S2: In-Solution Isoelectric Focussing of the Skeletal Muscle Proteome. Proteins were separated according to isoelectric point and each fraction further separated by 1D-SDS-PAGE; Figure S3: Protein Extraction for FASP Analysis. Proteins were solubilised prior to FASP analysis. Panel A shows the skeletal muscle soluble proteome used for standard 1D-SDS-PAGE with the extracted proteome for FASP analysis shown in Panel B.
