# Supplementary material for: Comparing Simplification Strategies for the Skeletal Muscle Proteome
Source: Proteomes. 2016 Mar 2;4(1):10. doi: 10.3390/proteomes4010010 (PMC5217366; doi:10.3390/proteomes4010010)
Supplement: Supplementary file 1 [file proteomes-04-00010-s001.zip › proteomes-04-00010-suppl/proteomes-04-00010-supplmentary.pdf]

Supplementary Materials: Comparing Simplification Strategies for the Skeletal Muscle Proteome

Bethany Geary, Iain S. Young, Philip Cash, Phillip D. Whitfield, Mary K. Doherty

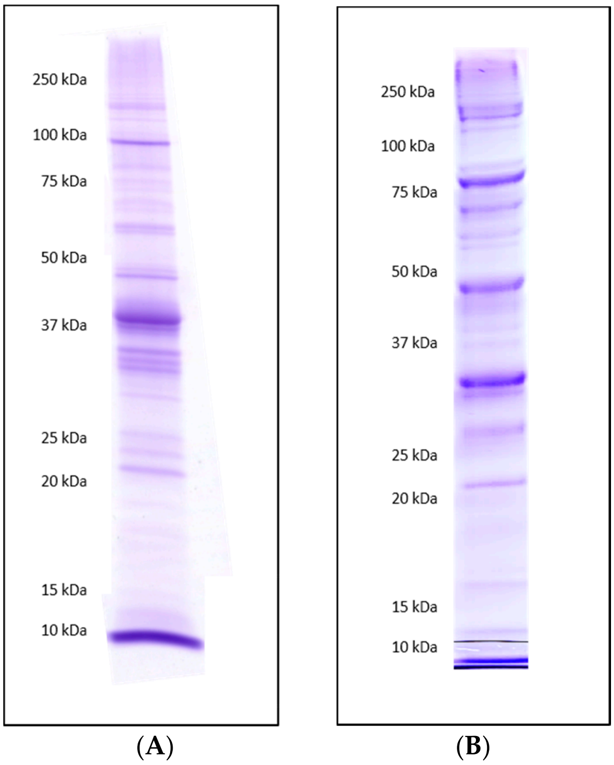

Figure S1. Visualisation of ProteoMiner Bead Equalisation. Proteins were subjected to equalisation using bead technology in order to reduce the dominance of highly abundant proteins. Panel **A** shows the pre-fractionated skeletal muscle soluble proteome with the simplified proteome in Panel **B**.

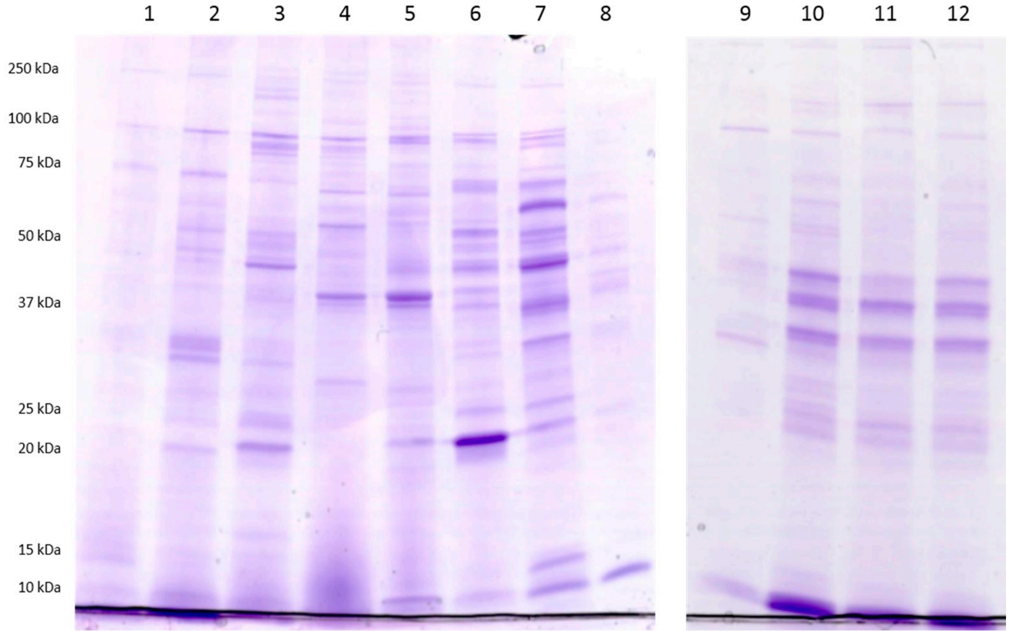

Figure S2. In-Solution Isoelectric Focussing of the Skeletal Muscle Proteome. Proteins were separated according to isoelectric point and each fraction further separated by 1D-SDS-PAGE.

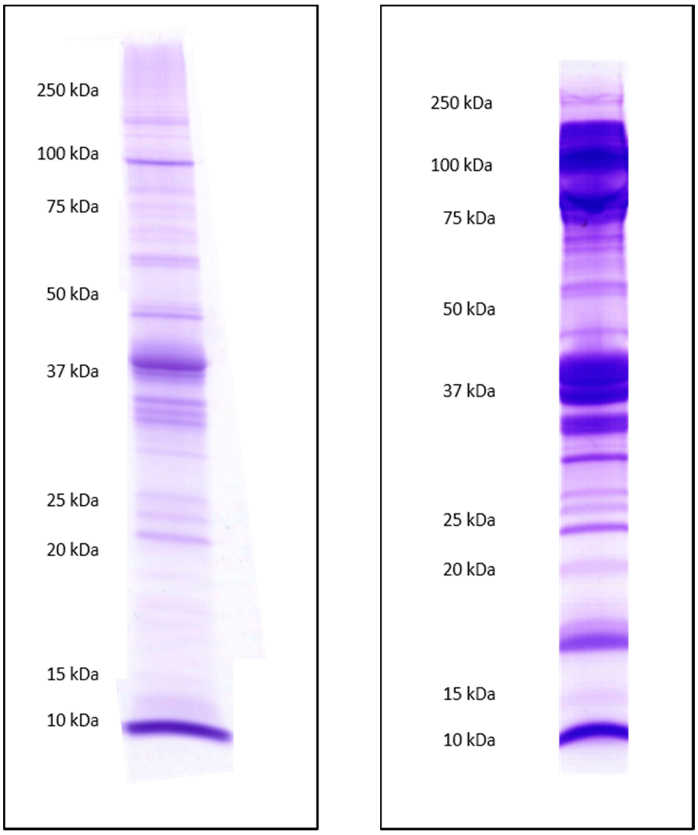

(**A**) (**B**)

Figure S3. Protein Extraction for FASP Analysis. Proteins were solubilised prior to FASP analysis. Panel **A** shows the skeletal muscle soluble proteome used for standard 1D-SDS-PAGE with the extracted proteome for FASP analysis shown in Panel **B**.
